# EFFECTIVENESS OF VISCOSUPPLEMENTATION IN THE TREATMENT OF HEMOPHILIC ARTHROPATHY: A SYSTEMATIC REVIEW

**DOI:** 10.1590/1413-785220233105e271857

**Published:** 2023-12-18

**Authors:** SAMILLY CONCEIÇÃO MAIA MARTINS, ERION DE ANDRADE, MAYARA BRANCO E SILVA, MARGARETH CASTRO OZELO, GUSTAVO CONSTANTINO DE CAMPOS, RODRIGO GONÇALVES PAGNANO

**Affiliations:** 1 Universidade Estadual de Campinas, Faculdade de Ciencias Medicas, Departamento de Ortopedia e Traumatologia, Campinas, SP, Brazil; 2 Universidade Estadual de Campinas, Faculdade de Ciencias Medicas, Departamento de Neurologia e Neurocirurgia, Campinas, SP, Brazil; 3 Universidade Estadual de Campinas, Centro de Hematologia e Hemoterapia, Unidade de Hemofilia “Claudio Luiz Pizzigatti Correa”, Campinas, SP, Brazil; 4 Universidade Estadual de Campinas, Faculdade de Ciencias Medicas, Departamento de Medicina Interna, Campinas, SP, Brazil

**Keywords:** Hyaluronic Acid, Viscosupplementation, Hemarthrosis, Pain Management, Arthropathy, Hemophilia, Ácido Hialurônico, Viscossuplementação, Hemartrose, Manejo da Dor, Artropatia, Hemofilia

## Abstract

**Objective:**

To describe the efficacy of using viscosupplementation in patients with hemophilic arthropathy (HA), on pain, limb functionality, and quality of life.

**Methods:**

A systematic review of the literature was performed following the PRISMA guidelines without limitations of language or year of publication. The search was performed on the following medical databases: PubMed, Cochrane Library, EMBASE, BVS/BIREME, Scopus, Web of Science, EBSCOhost, and PROQUEST in April 2020. The search used the following word: (hemophilia AND joint diseases) OR (haemophilic arthropathy OR hemophilic arthropathy) AND viscosupplementation.

**Results:**

The systematic review identified 127 articles, 10 of which were selected for data extraction and qualitative analysis. The 10 selected articles included 297 joints with HA in 177 hemophilic subjects. Our review showed positive results in alleviating pain and improving functional capacity, and quality of life. No major adverse effects were observed.

**Conclusion:**

There is a lack of scientific evidence regarding viscosupplementation with hyaluronic acid, but the results presented in this research suggest that it is an effective and safe therapeutic option to alleviate pain and improve functional capacity in patients with HA. ***Level of Evidence II, Systematic Review.***

## RESUMO

**Objetivo:**

Descrever o uso da viscossuplementação com ácido hialurônico em pacientes com artropatia hemofílica (HA), sua eficácia na dor, a funcionalidade do membro e a qualidade de vida após sua aplicação.

**Métodos:**

Revisão sistemática da literatura (RSL) que seguiu as diretrizes PRISMA, sem limitação de idioma ou ano de publicação. A pesquisa foi realizada em abril de 2020 nas seguintes bases de dados médicas: PubMed, Cochrane Library, EMBASE, BVS/BIREME, Scopus, Web of Science, EBSCOhost e ProQuest. A estratégia de pesquisa foi: (hemofilia AND joint disease) OR (artropatia hemofílica OU artropatia hemofílica) E viscossuplementação.

**Resultados:**

A RSL identificou 127 artigos, dos quais 10 foram selecionados para extração de dados e análise qualitativa. Os 10 artigos selecionados incluíram 297 articulações com AH em 177 indivíduos hemofílicos. Nossa revisão mostrou resultados positivos na melhora da dor, na capacidade funcional e na qualidade de vida. Não foram observados efeitos adversos importantes.

**Conclusão:**

A evidência científica atual a respeito da viscossuplementação com ácido hialurônico é escassa, mas os resultados apresentados nesta pesquisa sugerem que é uma opção terapêutica eficaz e segura para diminuir a dor e melhorar a capacidade funcional em pacientes com AH. **Nível de Evidência II, Revisão Sistemática.**

## INTRODUCTION

Hemophilia is a congenital bleeding disorder marked by frequent episodes of bleeding throughout life, particularly in the muscles and joints, called hemarthrosis.^1^ ^),(^ ^2^ Hemarthrosis is responsible for about 80% of all bleeding episodes. The direct action of iron and blood into joints leads to specific changes in the periarticular environment resulting in chronic synovitis, cartilage damage, and bone destruction, leading to irreversible changes. ^(^ ^3^ This process, called hemophilic arthropathy (HA), is multifactorial and a particular type of secondary osteoarthritis. ^(^ ^4^ ^),(^ ^5^ It usually affects young patients clinically presenting chronic pain, decreased range of motion, deformities, muscle atrophy, and functional impairment. ^(^ ^2^ ^),(^ ^6^ Therefore, HA has a high negative impact on the quality of life of patients with hemophilia. ^(^ ^3^ ^),(^ ^7^ ^),(^ ^8^ Hyaluronic acid is a molecule physiologically found in synovial fluid and cartilage matrix. ^(^ ^9^ Viscosupplementation, injection of hyaluronic acid, is an accepted treatment that can benefit patients with osteoarthritis through several different in vivo mechanisms by changing and decreasing the inflammatory and degenerative components, responsible for cartilage degeneration. ^(^ ^10^ ^),(^ ^11^ Among the described benefits are anti-inflammatory, anabolic, analgesic, and chondroprotective effects and their effect on the viscosity and elasticity of synovial fluid, thus reducing pain symptoms and contributing to lubrication, shock absorption, elasticity, hydration, and nutrition of joint tissues. ^(^ ^12^ ^),(^ ^13^ The clinical and biological similarity of the pathophysiology of osteoarthritis and HA led to the investigation of hyaluronic acid in patients with hemophilia that have HA. This study aimed to evaluate the efficacy of viscosupplementation in patients with hemophilic arthropathy regarding pain control, impact on limb functional capacity, and quality of life.

## METHODS

### Study selection

The search was performed in accordance with the Cochrane Model^14^ and the PRISMA (Preferred Reporting Items for Systematic Reviews and Meta-Analyses) recommendation. ^(^ ^15^ The “PICOT” methodology was used to define the clinical research issue and the search for evidence. The systematic search in eight electronic databases (PubMed, Cochrane Library, EMBASE, BVS/BIREME, Scopus, Web of Science, EBSCOhost, and PROQUEST) ^(^ ^13^ in April 2020. The research string was as follows: [medical subject descriptor terms (MeSH) and free terms] including (hemophilia AND “joint diseases”) OR (“hemophilic arthropathy” OR “haemophilic arthropathy”) AND viscosupplementation. To include the studies in the final analysis the following inclusion criteria were used: only studies on humans, randomized or non-randomized clinical trials, case-controlled studies, or case series, with no restrictions on year or language to minimize any risk of bias. Studies that included animal and in-vitro studies, literature reviews, case reports, duplicate papers, interviews, or comments were excluded. The retrieved studies were processed by reference management programs. Afterward, two independent reviewers (SCM and EJA) managed the remaining articles in the Rayyan program. Any discrepancies were resolved by discussion amongst the authors and consultations with the senior author (RCP) were made to revise the entire process.

### Outcomes of interest

The primary outcome assessed was clinical improvement in pain alleviation and function of the affected limb, and patient’s quality of life based on specific criteria and validated questionnaires. Visual Analog Pain Scale (VAS) was used to evaluate pain control. ^(^ ^16^ Regarding functional capacity, the Western Ontario and McMaster Universities Arthritis Index (WOMAC) was used, ^(^ ^17^ ^),(^ ^18^ and to assess patients quality of life, the 36-item Short-Form Survey (SF-36) scores was used. ^(^ ^19^ The secondary outcome was the occurrence of adverse effects.

### Statistical methods and analysis

As our search resulted in studies with different methodologies, including study designs, participants, interventions, and reported outcome measures it was not possible to perform a meta-analysis. Therefore, a qualitative synthesis of the data will be described.

## RESULTS

### Study search results

**Figure 1:**
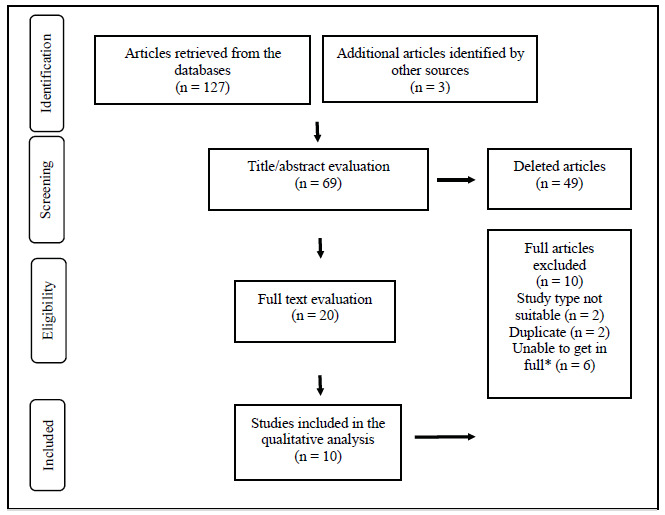
Research flowchart in the databases after applying the eligibility criteria.

The systematic search resulted in 127 articles. The retrieved studies were processed by reference management programs, where 61 papers were duplicates, and then automatically excluded. Afterward, two independent reviewers (SCM and EJA) managed the remaining articles in the Rayyan program, and three papers were excluded due to duplication. Also, 49 papers were excluded for the following reasons: inadequate study design (case reports, literature reviews, comments, interviews, or news); inappropriate population (not with hemophilic arthropathy), inappropriate intervention (studies that did not use intra-articular hyaluronic acid as a treatment method). The reviewers independently read the remaining 20 manuscripts in full and evaluated them according to the aforementioned eligibility criteria. Finally, we selected 10 articles for data extraction and qualitative analysis that evaluated intra-articular viscosupplementation in hemophilic patients, with regular follow-up with a hematologist and clotting factor replacement before performing the procedure. Figure 1 shows a flowchart outlining the selection process.

### Patient population

Altogether, the 10 selected articles evaluated the procedure in 297 joints in 177 subjects, with eight shoulders, 31 elbows, one hip, 181 knees, and 76 ankles. When reported, most subjects included in the studies were patients with severe hemophilia A. Table 1 summarizes the demographic data and clinical follow-up of the studies analyzed. The clinical protocol, therapeutic doses and interval of clinical evaluation of the results varied according to the administered product, location to be performed, and the availability of the patient, as shown in Table 2.

**Table 1:** The demographic data and clinical follow-up.

Study	Year	N	Age in years (mean)	BMI (mean)	Clinical Evaluation Timeframe	Follow-up range
Carulli et al. ^(^ ^7^	2012	46	39	26.7	0, 6, 12, 24 m	24-132 m
Carulli et al. ^(^ ^20^	2013	27	42	26.45	0, 6, 12, 24 m	60 m
Rezende et al. ^(^ ^21^	2015	14	23.7	NR	0, 1, 3, 6, 12 m	12 m
Zelada et al. ^(^ ^22^	2013	14	23.7	NR	0,1,3 m	3 m
Li et al. ^(^ ^23^	2019	11	38.8	25.4	0, 1, 3, 6, 12 m	6 m
Carulli et al. ^(^ ^24^	2020	14	45.8	NR	0, 1, 3, 6, 18 m	20 m
Li et al. ^(^ ^25^	2019	20	38.2	24.2	0, 1, 3, 6 m	6 m
Fernández-Palazzi et al. ^(^ ^26^	2002	25	29.7	NR	1-10 m	1-12 m
Wallny et al. ^(^ ^27^	2000	20	35-56	NR	0, 3, 24 m	26 m

BMI: body max index; m: month; NR: no results.

**Table 2:** Description of intervention protocols.

References	Description of Intervention Protocols
Carulli et al. ^(^ ^7^	3-5 intra-articular HAc administrations 1 to 4 weeks apart.
Carulli et al. ^(^ ^20^	5 Intra-articular low molecular weight HAc applications 2 weeks apart. 3 Applications of high molecular weight HAc 4 weeks apart
Rezende et al. ^(^ ^21^	In single intra-articular administration: joint lavage with 0.9% SF followed by infiltration with HAc (1 ampoule/2 ml) + triamcinolone (1 ml) diluted in ropivacaine (5 ml for knees and 2 ml for ankles, elbows, and shoulders).
Zelada et al. ^(^ ^22^	In single intra-articular administration: joint lavage with saline solution, followed by emptying and application of HAc (6 ml to the knee or 2 ml to the ankles, elbows, and shoulders) + triamcinolone (1 ml) + ropivacaine (5 ml to the knees or 1 ml to the ankles, elbows, and shoulders).
Li et al. ^(^ ^23^	5 intra-articular applications of 2.5 ml of HAc with a 1-week interval.
Carulli et al. ^(^ ^24^	3 intra-articular applications of HAc with monthly intervals in the knees and 2 applications with monthly intervals in the ankle.
Li et al. ^(^ ^25^	3 Intra-articular applications of HAc (2 ml) with weekly intervals,
Fernández-Palazzi et al. ^(^ ^26^	3 Intra-articular HAc administrations through standard portals, at weekly intervals.
Wallny et al. ^(^ ^27^	5 applications of HAc (01 ampoule of 20 mg) intra-articular, with a weekly interval.

### Clinical Outcomes

The clinical outcomes evaluated were pain and functional capacity, as summarized in Table 3.

**Table 3:** Clinical scores using the scales EVA, WOMAC, and SF-36 applied pre- and post-treatment with HAc.

STUDY	VAS		WOMAC		SF-36	
	Pre	Post	Pre	Post	Pre	Post
Carulli et al. ^(^ ^20^	5.52	1 m: 2.45	64.45	6 m: 21.2	52.57	1 m: NR
		12 m: 2.98		12 m: 54.2		2 m: NR
		24 m: 3.12		24 m: 56.6		3 m: NR
				36 m: 56.8		6 m: 72.5
						12 m: 72.5
						24 m: 66.1
						36 m: 47.4
Rezende et al. ^(^ ^21^	4.57	1 m: 3.56	34.4	1 m: 24.1	NR	1 m: NR
		3 m: 4.2		2 m: NR		2 m: NR
		6 m: 4.23		3 m: 23.5		3 m: NR
		12 m: 3.82		6 m: 23.5		6 m: NR
				12 m: 22,4		12 m: NR
				24 m: NR		24 m: NR
				36 m: NR		36 m: NR
Zelada et al. ^(^ ^22^	44.6	1 m: 4.4	38,4	1 m: 23.5	32	1 m: 62.4
		2 m: NR		2 m: NR		2 m: NR
		3 m: 4.6		3 m: 26.5		3 m: 92.4
		6 m: NR		6 m: NR		6 m: NR
		12 m: NR		12 m: NR		12 m: NR
				24 m: NR		24 m: NR
				36 m: NR		36 m: NR
Li et al. ^(^ ^23^	Knee: 4,1	1 m: 1.8**	38.3	1 m: 19.1	54.4	1 m: 58.5
		2 m: 1.6**		2 m: 21.3		2 m: 63.5
		3 m: 2.3**		3 m: 27.1		3 m: 63.3
		6 m: NR		6 m: 35.8		6 m: 58.3
		12 m: NR		12 m: NR		12 m: NR
				24 m: NR		24 m: NR
				36 m: NR		36 m: NR
Carulli et al. ^(^ ^24^	8	1 m: 1*	NR	1 m: NR	NR	1 m: NR
		2 m: NR		2 m: NR		2 m: NR
		3 m: NR		3 m: NR		3 m: NR
		6 m: NR		6 m: NR		6 m: NR
		12 m: NR		12 m: NR		12 m: NR
				24 m: NR		24 m: NR
				36 m: NR		36 m: NR
Li et al. ^(^ ^25^	Knee: 5.7	1 m: 2.7	38.1	1 m: 22.3	48.8	1 m: 58.8
		2 m: 1.8		2 m: 21.3		2 m: 63.2
		3 m: 2.5		3 m: 24.8		3 m: 64.8
		6 m: 3.2		6 m: 26		6 m: 60.6
		12 m: NR		12 m: NR		12 m: NR
				24 m: NR		24 m: NR
				36 m: NR		36 m: NR
Fernández-Palazzi et al. ^(^ ^26^	Shoulder: 7,67	1 m: 3.7	NR	1 m: NR	NR	1 m: NR
	Elbow: 10	2 m: NR		2 m: NR		2 m: NR
	Knee: 8.47	3 m: NR		3 m: NR		3 m: NR
	Ankle: 8	6 m: NR		6 m: NR		6 m: NR
		12 m: NR		12 m: NR		12 m: NR
				24 m: NR		24 m: NR
				34 m: NR		36 m: NR
Wallny et al. ^(^ ^27^	Knee: 5.4	1 m: 4.7	NR	1 m: NR	NR	1 m: NR
		2 m: NR		2 m: NR		2 m: NR
		3 m: NR		3 m: NR		3 m: NR
		6 m: NR		6 m: NR		6 m: NR
		12 m: NR		12 m: NR		12 m: NR
				24 m: NR		24 m: NR
				36 m: NR		36 m: NR

VAS: Visual Analog Pain Scale; WOMAC: The Western Ontario and McMaster Universities Osteoarthritis Index; SF-36: 36-item Short-Form Survey; m: month; NR: no results.

#### Pain assessment

Pain was assessed by the VAS^16^ with a pre-procedure mean score of 5.6 (range: 4.1-8.7). Carulli et al., ^(^ ^7^ ^),(^ ^20^ in a long-term follow-up study demonstrated maximum benefit six months after the intervention compared to the pre-intervention (p < 0.05). Carulli et al. ^(^ ^7^ reported in their series, including 46 patients with hemophilia, that eight out of 10 evaluated elbows showed marked alleviation of pain, with only two patients needing additional analgesia or complementary physical therapy to control pain. The same author reported 15 out of 24 joints assessed had improvement on pain scores in the knee. Of 25 patients whose ankles were evaluated only three required analgesia or physical therapy to control pain, and one was indicated for ankle arthroplasty due to poor improvement. The same author concluded that viscosupplementation was able to delay aggressive treatment for up to 2 to 4 years after the first cycle with 91.4% of patients exhibiting good results. However, in a different study, Carulli et al., ^(^ ^20^ without differing joints, observed that all patients found pain alleviation in the short term compared to the pre-treatment assessment (p < 0.05) up to the first year and with a subsequent gradual decline, nonetheless still better than pre-intervention values.

Fernández-Palazzi et al., ^(^ ^26^ observed complete pain relief in 13.7% of the injected joints and partial improvement in 62%, which means that 75% of the results were classified as excellent or good outcomes. Three-quarters of the patients improved, and only 10.3% were considered to have a poor outcome, wherein there was no improvement in the joint condition, requiring another procedure.

Wallny et al., ^(^ ^27^ reported that the VAS^16^ for the subjective experience of pain dropped from 5.4 to 3.8 points, improving after three months in 70% of their patients. They also observed that the positive effect of viscosupplementation was maintained for up to two years in half of the patients.

Li et al., ^(^ ^23^ obtained a significant reduction in pain from hemophilic arthropathy of the knee, observed for up to six months (p < 0.01). The authors followed the maximum benefit two months after injection.

#### Functional capacity and quality of life

Functional capacity was evaluated using the WOMAC score, that is a disease-specific measure to evaluate limb function in arthritis and arthropathies with values from 0 (best) to 100 (worst). Carulli et al., ^(^ ^20^ reported pre-treatment mean value of 64.45 and observed a maximum benefit in six months (mean value = 21.2) with a gradual increase in their values (mean value of 56.8 in 36 months). Subsequently, Rezende et al. ^(^ ^21^ observed an average decrease of 8.29 points compared to pre-treatment after one month. Zelada et al., ^(^ ^22^ in a study with 3 months of follow-up, found a greater difference in the total value in one month, up to minus 14.7 points, mainly at the expense of function improvement, with an average decrease of 11.4 points (p < 0.05). The other papers selected did not assess WOMAC scores.

Among the 10 papers, quality of life assessment using the SF-36 was reported in five papers. The SF-36 is a 36-item assessment tool that aims to perform a generic measure of health status evaluating physical functioning, social functioning, and role limitation due to physical health or mental problems, with higher scores indicating better health-related quality of life. Carulli et al., ^(^ ^20^ presented a pre-treatment mean score of 52.57 points and a significant difference with improved functional capacity compared to the pre-intervention at six months (mean score = 72.5, p < 0,05), reaching better levels and associated with substantially positive effects in the long-term follow-up at 36 months (mean score = 47.4). It can be noted that an increase in the self-reported questionnaire values were observed after three months of treatment, followed by a slow decline over time. In Li et al., ^(^ ^23^ the total result was not statistically significant with pre-treatment mean scores of 54.4 and the largest increase in scores was observed in 2 months with mean score of 63.5. In another study, Li et al. ^(^ ^25^ described a mean pre-treatment score of 68.8 and the stronger benefit was recorded 3 months post-treatment with mean score of 64.8 with scores slightly decreasing at the 6-month follow-up (mean score = 60.6) In the studies whose scores were stratified by the components of SF-36, it was observed that most of the improvement in scores was due to the mental health component ^7^ ^), (^ ^20^ ^), (^ ^22^ ^), (^ ^23^ ^), (^ ^25^ ^), (^ ^28^ ^).^

### Adverse effects and procedure complications

The viscosupplementation in the evaluated studies showed that the patients had good tolerance to the intra-articular injections. Some minor and transient adverse effects at the injection site, such as pain after injection and local bruising, have been reported by Li et al. ^(^ ^25^ In this review, there was no joint bleeding related to the intra-articular application of hyaluronic acid, post-procedure infection, or acute inflammation. The studies in this review reported no major adverse effects.

## DISCUSSION

As the life expectancy of patients with hemophilia has increased, the management of its consequences, such as pain and decreased functional capacity, has become a central issue in the comprehensive treatment because of its impact on patients’ quality of life. Clinical data of patients with hemophilia shows that joint pain is the most common painful manifestation and a substantial problem, where patients often feel that their pain has been sub-optimally managed despite medical treatment. ^(^ ^24^ ^),(^ ^25^ ^),(^ ^28^ ^),(^ ^29^

Pain management strategies for patients with hemophilia involve a multimodal approach, focusing on physical and psychological aspects, and suggesting a gradual process according to pain intensity. Whenever possible, the underlying condition should be treated in a staggered manner, such as by physical therapy, analgesic and anti-inflammatory treatment, radioisotope synovectomy, and surgical interventions. ^(^ ^30^

Analgesic medical therapy in hemophilia patients shows additional challenges due to the need for long-term use, comorbidities, and the potential of some medications to increase the risk of bleeding. ^(^ ^31^ Several studies confirm that intra-articular hyaluronic acid is effective in treating osteoarthritis and supports its use. ^(^ ^32^ ^), (^ ^33^ ^), (^ ^34^ ^), (^ ^35^ ^), (^ ^36^ ^), (^ ^37^ ^), (^ ^38^ Rodrigues-Merchan, ^(^ ^39^ in a literature review on intra-articular injections of hyaluronic acid in the hemophilic knee, emphasizes the similarities and differences between primary osteoarthritis (OA) and HA and provide a rationale for defending the use of viscosupplementation in patients with hemophilia. The author emphasizes that, as in OA, there is joint destruction associated with pain, loss of range of motion, deformities, and functional incapacity of the affected limb. However, in hemophilic arthropathy, these characteristics are more intense and occur at an earlier age. Fernández-Palazzi et al., ^(^ ^26^ are among the first authors to propose the efficacy and safety of hyaluronic acid administration in chronic hemophilic arthropathy. In their study, with a mean follow-up of two years, most patients had positive and persistent effects, such as pain relief and improvements in range of motion and functional capacity. Only 10% of patients were considered to have a poor outcome requiring new treatments. The authors’ main conclusion was that viscosupplementation is effective and a better physiological treatment than corticosteroid therapy without the harmful effects on the articular cartilage known to be caused by the latter.

Carulli et al., ^(^ ^7^ proposed viscosupplementation as a primary approach to HA. With changes in lifestyle and rehabilitation, it can be recommended for all patients with hemophilia with initial radiological signs of arthropathy associated with pain and functional impairment. The authors showed that injections with hyaluronic acid were positive, in the short term, in modulating pain and functional capacity in the knees, ankles, and elbows. An average six-year follow-up showed a reduction in the degeneration of joint function. In their series, some patients required more than two injections over the years, with a positive and lasting impact on pain control and range of motion, reducing the need for a more invasive approach. In two other papers from the same group, Carulli et al., ^(^ ^20^ ^),(^ ^24^ showed the same positive results when comparing HA patients treated with viscosupplementation to a nontreated hemophilic population; they also suggest the use and the benefits of hyaluronic acid for severe arthropathy with the intention to postpone an invasive procedure. More than half of hemophilic patients with arthropathy report mobility problems, especially those with bleeding despite prophylaxis. ^(^ ^28^ The WOMAC was developed in the early-1980s as a disease-specific measure for lower limb arthritis and arthropathy. ^(^ ^17^ ^),(^ ^18^ Our selected studies^20^ ^)-(^ ^23^ verified that viscosupplementation can improve functional capacity, based on the WOMAC score, in the short term with a subsequent slow decline in the scores, but still showing better values than pre-treatment, especially those related to joint stiffness and range of motion, with more persistent positive effects on these areas. In agreement with the available literature, ^(^ ^39^ we did not observe major adverse effects in the evaluated studies.

Hoorfar and Mobaraky^40^ used the SF-36 tool to assess their patients. The authors reported that patients with hemophilia and HA have a self-perceived physical disability with especially low scores in physical domains related to pain. Zelada et al., ^(^ ^22^ were able to verify the same results. When analyzing the post-treatment scores, the aforementioned studies reported that the physical component of the SF-36 showed improvement by the procedure, but the mental component of the SF-36 was the one that improved the most, mainly after three months of the procedure.

Despite the beneficial results being more expressive in the short term, especially in the first six months, it is essential to highlight that for those living with hemophilia, less invasive procedures to the musculoskeletal system are especially interesting. Therefore, as a less invasive procedure, viscosupplementation provides benefits such as pain relief and joint protection, with improved load distribution and reduced impact. Thus, especially in the studied population, it can enable adequate rehabilitation and serve the purpose of a less invasive treatment, adding to an improvement in the long-term quality of life.

Our study has some limitations. The quality of the studies varied, with most being marked by low-level evidence as descriptive or case series (level of evidence III or IV). Also, the studies selected showed marked methodological variations, including study designs, participants, intervention protocols, and reported outcome

measures making the statistical analysis impossible. Therefore, we describe the studies, their results, applicability, and limitations in the qualitative synthesis. Concerning the results of the review, this article highlights the scarcity of publications on hemophilic arthropathy and the consequent restriction in data analysis.

## CONCLUSION

According to the available literature, viscosupplementation can be a useful therapeutic option in hemophilic arthropathy, with positive results in alleviating pain and improving functional capacity and quality of life, especially in the first six months, and with no major adverse effects. Those results are especially important in this specific population that presents a fast disease progression at an early age.

## References

[1] Shopnick RI, Brettler DB (1996). Hemostasis a practical review of conservative and operative care. Clin Orthop Relat Res.

[2] Rodriguez-Merchan EC, de la Corte H, Rodriguez-Merchan EC, Goddard NJ, Lee CS (2008). Musculoskeletal aspects of haemophilia.

[3] Stephensen D, Tait RC, Brodie N, Collins P, Cheal R, Keeling D (2009). Changing patterns of bleeding in patients with severe haemophilia A. Haemophilia.

[4] Thorat T, Neumann PJ, Chambers JD (2018). Hemophilia burden of disease a systematic review of the cost-utility literature for hemophilia. J Manag Care Spec Pharm.

[5] Melchiorre D, Manetti M, Matucci-Cerinic M (2017). Pathophysiology of hemophilic arthropathy. J Clin Med.

[6] Lafeber FPJG, Miossec P, Valentino LA (2008). Physiopathology of haemophilic arthropathy. Haemophilia.

[7] Carulli C, Civinini R, Martini C, Linari S, Morfini M, Tani M, Innocenti M (2012). Viscosupplementation in haemophilic arthropathy a long-term follow-up study. Haemophilia.

[8] Krasuska M, Riva S, Fava L, von Mackensen S, Bullinger M (2012). Linking quality-of-life measures using the International Classification of Functioning, Disability and Health and the International Classification of Functioning, Disability and Health-Children and Youth Version in chronic health conditions the example of young people with hemophilia. Am J Phys Med Rehabil.

[9] Fraser JR, Clarris BJ, Baxter E (1979). Patterns of induced variation in the morphology, hyaluronic acid secretion, and lysosomal enzyme activity of cultured human synovial cells. Ann Rheum Dis.

[10] Håkansson L, Hällgren R, Venge P (1980). Regulation of granulocyte function by hyaluronic acid In vitro and in vivo effects on phagocytosis, locomotion, and metabolism. J Clin Invest.

[11] Punzi L, Schiavon F, Cavasin F, Ramonda R, Gambari PF, Todesco S (1989). The influence of intra-articular hyaluronic acid on PGE2 and cAMP of synovial fluid. Clin Exp Rheumatol.

[12] Gibbs DA, Merrill EW, Smith KA, Balazs EA (1968). Rheology of hyaluronic acid. Biopolymers.

[13] Sun SF, Chou YJ, Hsu CW, Chen WL (2009). Hyaluronic acid as a treatment for ankle osteoarthritis. Curr Rev Musculoskelet Med.

[14] Cumpston M, Li T, Page MJ, Chandler J, Welch VA, Higgins JP, Thomas J (2019). Updated guidance for trusted systematic reviews a new edition of the Cochrane Handbook for Systematic Reviews of Interventions. Cochrane Database Syst Rev.

[15] Liberati A, Altman DG, Tetzlaff J, Mulrow C, Gøtzsche PC, Ioannidis JPA (2009). The PRISMA statement for reporting systematic reviews and meta-analyses of studies that evaluate health care interventions explanation and elaboration. J Clin Epidemiol.

[16] Downie WW, Leatham PA, Rhind VM, Wright V, Branco JA, Anderson JA (1978). Studies with pain rating scales. Ann Rheum Dis.

[17] Bellamy N, Buchanan WW, Goldsmith CH, Campbell J, Stitt LW (1988). Validation study of WOMAC a health status instrument for measuring clinically important patient relevant outcomes to antirheumatic drug therapy in patients with osteoarthritis of the hip or knee. J Rheumatol.

[18] Faik A, Benbouazza K, Amine B, Maaroufi H, Bahiri R, Lazrak N (2008). Translation and validation of Moroccan Western Ontario and McMaster Universities (WOMAC) osteoarthritis index in knee osteoarthritis. Rheumatol Int.

[19] Patel AA, Donegan D, Albert T (2007). The 36-item short form. J Am Acad Orthop Surg.

[20] Carulli C, Matassi F, Civinini R, Morfini M, Tani M, Innocenti M (2013). Intra-articular injections of hyaluronic acid induce positive clinical effects in knees of patients affected by haemophilic arthropathy. Knee.

[21] Rezende MU, Rosa TBC, Pasqualin T, Frucchi R, Okazaki E, Villaça PR (2015). Subjective results of joint lavage and viscosupplementation in hemophilic arthropathy. Acta Ortop Bras.

[22] Zelada F, Almeida AM, Pailo AF, Bolliger R, Okazaki E, Rezende MU (2013). Viscosupplementation in patients with hemophilic arthropathy. Acta Ortop Bras.

[23] Li TY, Wu YT, Chen LC, Cheng SN, Pan RY, Chen YC (2019). An exploratory comparison of single intra-articular injection of platelet-rich plasma vs hyaluronic acid in treatment of haemophilic arthropathy of the knee. Haemophilia.

[24] Carulli C, Rizzo AR, Innocenti M, Civinini R, Castaman G, Innocenti M (2020). Viscosupplementation in symptomatic haemophilic arthropathy of the knee and ankle experience with a high molecular weight hyaluronic acid. Haemophilia.

[25] Li TY, Wu YT, Chen LC, Cheng SN, Pan RY, Chen YC (2019). Efficacy, safety, and synovial effects of intra-articular hyaluronic acid in treating recalcitrant hemophilic arthropathy of knee joint. J Med Sci.

[26] Fernández-Palazzi F, Viso R, Boadas A, Ruiz-Sáez A, Caviglia H, De Bosch NB (2002). Intra-articular hyaluronic acid in the treatment of haemophilic chronic arthropathy. Haemophilia.

[27] Wallny T, Brackmann HH, Semper H, Schumpe G, Effenberger W, Hess L, Seuser A (2000). Intra-articular hyaluronic acid in the treatment of haemophilic arthropathy of the knee Clinical, radiological and sonographical assessment. Clinical, radiological and sonographical assessment. Haemophilia.

[28] Witkop M, Lambing A, Divine G, Kachalsky E, Rushlow D, Dinnen J (2012). A national study of pain in the bleeding disorders community a description of haemophilia pain. Haemophilia.

[29] Forsyth AL, Witkop M, Lambing A, Garrido C, Dunn S, Cooper DL, Nugent DJ (2015). Associations of quality of life, pain, and self-reported arthritis with age, employment, bleed rate, and utilization of hemophilia treatment center and health care provider services results in adults with hemophilia in the HERO study. Patient Prefer Adherence.

[30] Riley RR, Witkop M, Hellman E, Akins S (2011). Assessment and management of pain in haemophilia patients. Haemophilia.

[31] van Vulpen LFD, Holstein K, Martinoli C (2018). Joint disease in haemophilia pathophysiology, pain and imaging. Haemophilia.

[32] Carrabba M, Paresce E, Angelini M, Zamboni AM, Bragantini A, Paissan A (1992). The intra-articular treatment of osteoarthritis of the knee A comparative study between hyaluronic acid (Hyalgan(r)) and orgotein. Eur J Rheumatol Inflamm.

[33] Dougados M, Nguyen M, Listrat V, Amor B (1993). High molecular weight sodium hyaluronate (hyalectin) in osteoarthritis of the knee a 1 year placebo-controlled trial. Osteoarthritis Cartilage.

[34] Jones AC, Pattrick M, Doherty S, Doherty M (1995). Intra-articular hyaluronic acid compared to intra-articular triamcinolone hexacetonide in inflammatory knee osteoarthritis. Osteoarthritis Cartilage.

[35] Graf J, Neusel E, Schneider E, Niethard FU (1993). Intra-articular treatment with hyaluronic acid in osteoarthritis of the knee joint a controlled clinical trial versus mucopolysaccharide polysulfuric acid ester. Clin Exp Rheumatol.

[36] Dahlberg L, Lohmander LS, Ryd L (1994). Intraarticular injections of hyaluronan in patients with cartilage abnormalities and knee pain A one-year double-blind, placebo-controlled study. Arthritis Rheum.

[37] Gigante A, Callegari L (2011). The role of intra-articular hyaluronan (Sinovial) in the treatment of osteoarthritis. Rheumatol Int.

[38] Foti C, Cisari C, Carda S, Giordan N, Rocco A, Frizziero A, Della Bella G (2011). A prospective observational study of the clinical efficacy and safety of intra-articular sodium hyaluronate in synovial joints with osteoarthritis. Eur J Phys Rehabil Med.

[39] Rodriguez-Merchan EC (2012). Intra-articular injections of hyaluronic acid (viscosupplementation) in the haemophilic knee. Blood Coagul Fibrinolysis.

[40] Hoorfar H, Mobaraky G (2006). Quality of life in severe hemophilia in Esfahan. Haemophilia.

